# Bayesian composite quantile regression for the single-index model

**DOI:** 10.1371/journal.pone.0285277

**Published:** 2023-05-10

**Authors:** Xiaohui Yuan, Xuefei Xiang, Xinran Zhang

**Affiliations:** 1 School of Mathematics, Jilin University, Changchun, China; 2 School of Mathematics and Statistics, Changchun University of Technology, Changchun, China; New York University Grossman School of Medicine, UNITED STATES

## Abstract

By using a Gaussian process prior and a location-scale mixture representation of the asymmetric Laplace distribution, we develop a Bayesian analysis for the composite quantile single-index regression model. The posterior distributions for the unknown parameters are derived, and the Markov chain Monte Carlo sampling algorithms are also given. The proposed method is illustrated by three simulation examples and a real dataset.

## 1 Introduction

The single-index model (SIM) is one of the most popular semiparametric models in statistics, econometrics and psychometrics. In recent years, there are extensive researches on fitting SIMs using kernel, local linear and average derivatives methods. Among them, two most popular methods are the average derivative (ADE) method (Powell et al. [1], H*ä*rdle and Stoker [2], H*ä*rdle et al. [3]) and the minimum average variance estimation (MAVE) method (Xia and H*ä*rdle [4], Chen et al. [5], Zhao and Feng [6]).

Most of the above methods were based on conditional mean model. As a useful supplement to mean regression, quantile regression (Koenker and Bassett [7]) produced a more complete description of the conditional response distribution. Wu et al. [8] proposed a practical algorithm based on the local linear approach to estimate the nonparametric link function and the quantile regression coefficient. Lv et al. [9] proposed a quantile regression estimation for partially linear single-index model by minimizing the average quantile loss estimation method and multidimensional kernel estimation method. Jiang and Qian [10] used the kernel method to estimate the unknown function and developed a back-fitting algorithm for single-index model based on quantile regression. Xu et al. [11] investigated quantile regression (QR) estimation for single-index QR models when the response was subject to random left truncation. The asymptotic properties for the proposed QR estimates of the index parameter and unknown link function were both obtained. Although, the quantile regression is more robust than the mean regression, sometimes, quantile regression may still loss some efficiency. In order to safeguard quantile regression against potiential efficiency loss, the composite quantile regression (CQR) combining information over different quantiles becomes more and more popular. Intuitively, the CQR could provide an effective estimation for the SIM. Jiang et al. [12] suggested a back-fitting CQR algorithm for SIM. Later, a two-step CQR estimation procedure for SIMs was proposed by Jiang et al [13]. Jiang et al [14] proposed a weighted CQR estimation for SIMs. Liu et al. [15]considered weighted composite quantile estimation of the single-index model with missing covariates at random. Jiang and Yu [16] extended the non-iterative composite quantile regression methods for single-index models to the analysis of massive datasets via a divide-and-conquer strategy. The proposed approach significantly reduced the computing time and the required primary memory. Song et al. [17] focused on the estimators of the parameters and the unknown link function for the single-index model in a high-dimensional situation. The SCAD and Laplace error penalty (LEP)-based penalized composite quantile regression estimators, which could realize variable selection and estimation simultaneously.

In the past few years, there were extensive researches about frequentist estimation of single-index regression models, but Bayesian approach is also a useful statistical analysis tool. For Bayesian approach of nonparametric link function, Antoniadis et al. [18] approximated the link function by B-splines and adopted regularization with generalized cross validation to avoid over-fitting. Wang [19] estimated the index vector and the link function by free-knot splines. Choi et al. [20] considered a Gaussian process regression (GPR) approach to analysing a SIM from the Bayesian perspective, the proposed approach broadened the scope of the applicability of the SIM as well as the GPR. In addition, they discussed the theoretical aspect of the proposed method in terms of posterior consistency. Gramacy and Lian [21] developed a SIM for parsimonious multidimensional nonlinear regression by combining parametric projection with univariate nonparametric regression models. They showed that a particular GP formulation was simple to work with and ideal as an emulator for some types of computer experiment. Hu et al. [22] used a Gaussian process prior for the unknown nonparametric link function and a Laplace distribution for the index vector, which demonstrated the advantages of the Bayesian method compared with the frequentist approach. Liu and Liang [23] investigated single-index quantile regression with missing observation based on Bayesian method, while using spline approximation for the link function. They constructed quasi-posterior distribution of the index vector based on asymmetric Laplace likelihood with missing observation, and established asymptotically normality of the posterior estimator of the index parameters.

As far as we know, there are few work to consider composite quantile regression single-index model based on Bayesian approach. Therefore, this paper consider the Bayesian technique to study the composite quantile regression for single-index models. By using a Gaussian process prior and a location-scale mixture representation of the asymmetric Laplace distribution, we develop a Bayesian analysis for the composite quantile single-index regression model. The posterior distributions for the unknown parameters are derived, and the Markov chain Monte Carlo sampling algorithms are also given. Favorable performance is illustrated on two simulation examples and the real dataset.

This article is organized as follows: In Section 2, we introduce the composite quantile regression for single-index model firstly. Then, the details of our hierarchical Bayesian composite quantile single-index regression model are provided. We execute the posterior sampling algorithm by a more efficient partially collapsed sampler. Note that the link function is integrated out when drawing samples of the index vector. Some detailed derivative processes are showed in detail in the S1 Appendix. Then, numerical illustrations including simulation studies and the real data is presented in Section 3 and section 4. We conclude the paper with a discussion in Section 5.

## 2 Bayesian hierarchical model

### 2.1 Model structure

In the composite quantile single-index regression model, for independent and identically distributed pairs (***x***_*i*_, *y*_*i*_), *i* = 1, 2, …, *n*, we assume

$$\begin{array}{c} y_{i}=\eta \left({x_{i}}^{T}\beta \right)+\varepsilon_{i}, \end{array}$$ (1)

where *y*_*i*_ is the response, ***x***_*i*_ = (*x*_*i*1_, *x*_*i*2_, …, *x*_*ip*_)^*T*^ is the p-dimensional covariate, *η*(⋅) is an unknown link function, ***β*** = (*β*_1_, *β*_2_, …, *β*_*p*_)^*T*^ is an unknown parameter vector, and ***ε***_*i*_ is the error. The aim of this paper is to simultaneously estimate ***β*** and *η*(⋅).

According to Zou and Yuan [24], we denote 0 < *τ*_1_ < *τ*_2_ < ⋯ < *τ*_*M*_ < 1, the parameter estimators of composite quantile single-index regression is defined as:

$$\begin{array}{c} \left(\hat{\alpha_{1}},\hat{\alpha_{2}},\ldots ,\hat{\alpha_{M}},\hat{\beta }\right)=\underset{\alpha_{1},\alpha_{2},\ldots ,\alpha_{M},\beta }{\text{min}}\;\sum_{m=1}^{M}\{\sum_{i=1}^{n}\rho_{\tau_{m}}\left[y_{i}-\alpha_{m}-\eta \left({x_{i}}^{T}\beta \right)\right]\}, \end{array}$$ (2)

where *m* = 1, 2, ⋯, *M*, $\tau_{m}=\frac{m}{M+1}$, *α*_*m*_ is the intercept at the quantile level *τ*_*m*_ and $\rho_{\tau_{m}}\left(t\right)=t\left(\tau_{m}-I\left(t<0\right)\right)$ is the check loss function at *τ*_*m*_, *I*(⋅) denotes the indicator function.

### 2.2 Hierarchical bayesian modelling

At the *τ*-th quantile, we model the residual errors by the asymmetric Laplace distribution (ALD, Yu and Moyeed [25], Geraci and Bottai [26], Luo et al. [27]). More specifically, the probability distribution of *y* given *μ* = *η*(***x***^*T*^***β***) is

$$\pi \left(y|\mu ,\sigma \right)=\frac{\tau (1-\tau )}{\sigma }\;\text{exp}\;\{-\frac{1}{\sigma }\rho_{\tau }\left(y-\mu \right)\},$$

where the quantile level *τ* is the skewness parameter in the distribution, *μ* is the location parameter, and *σ* is the scale parameter. Let $\mu_{i}=\eta \left(x_{i}^{T}\beta \right)$, and ***y*** = (*y*_1_, *y*_2_, …, *y*_*n*_)^*T*^, the conditional distribution for the observations is

$$\pi \left(y|\beta ,\eta ,\sigma \right)=\frac{\tau^{n}{\left(1-\tau \right)}^{n}}{\sigma^{n}}\;\text{exp}\;\{-\frac{1}{\sigma }\sum_{i=1}^{n}\rho_{\tau }[y_{i}-\eta \left(x_{i}^{T}\beta \right)]\}.$$

Thus, the minimization objective function in (2.2) can be rewritten as the likelihood function of a composite quantile single-index regression:

$$\begin{array}{c} \pi \left(y|\beta ,\eta ,\sigma ,\alpha_{m}\right)=\prod_{m=1}^{M}\frac{\tau_{m}^{n}{\left(1-\tau_{m}\right)}^{n}}{\sigma^{n}}\;\text{exp}\;\{-\frac{1}{\sigma }\sum_{i=1}^{n}\rho_{\tau_{m}}[y_{i}-\alpha_{m}-\eta \left({x_{i}}^{T}\beta \right)]\}. \end{array}$$ (3)

However, there are some troubles in handling directly the above likelihood for Bayesian inference. A location-scale mixture representation of the ALD (Kozumi and Kobayashi [28]) is helpful to deal with this difficulty. We can write the observations satisfying model (1) as

$$\begin{array}{c} y_{i}=\alpha_{m}+\eta \left({x_{i}}^{T}\beta \right)+\left(1-2\tau_{m}\right)e_{im}+{\left(2e_{im}\sigma \right)}^{1/2}\varepsilon_{i}, \end{array}$$ (4)

where *e*_*im*_ ∼ exp(*τ*_*m*_(1 − *τ*_*m*_)*σ*^−1^), *ε*_*i*_ is a standard normal random variable, which is independent of *e*_*im*_. For proposed Bayesian composite quantile regression for single-index, the model (4) can be written as:

$$y_{i}|\eta ,\alpha_{m},\beta ,e_{im}∼N\left(\alpha_{m}+\eta \left({x_{i}}^{T}\beta \right)+\left(1-2\tau_{m}\right)e_{im},2\sigma e_{im}\right).$$

Thus, the complete likelihood function of model (3) based on observations (***x***, ***y***) can be written as:

$$\begin{array}{ccc} \pi (y_{i}|\beta ,\eta_{n},e_{im},\alpha_{m}) & = & {\left(4\pi \sigma \right)}^{-\frac{Mn}{2}}{\left(\prod_{m=1}^{M}\prod_{i=1}^{n}e_{im}\right)}^{-\frac{1}{2}}\\ & & \times \;\text{exp}\;\{-\frac{1}{4}\sum_{i=1}^{n}\sum_{m=1}^{M}\frac{1}{e_{im}}{\left[y_{i}-\alpha_{m}-\eta \left({x_{i}}^{T}\beta \right)-\left(1-2\tau_{m}\right)e_{im}\right]}^{2}\}. \end{array}$$

Followng Choi et al. [20] and Gramacy and Lian [21], we model the unknown link function *η*(⋅) by a Gaussian process prior distribution. More specifically, *η* is a Gaussian process prior with zero mean and a squared-exponential covariance function, that is

$$\eta ∼GP\left(0,C\left(\cdot ,\cdot \right)\right),C\left(\nu ,\nu^{\prime }\right)=\gamma \;\text{exp}\;\{-\frac{{\left(\nu -\nu^{\prime }\right)}^{2}}{d}\},$$

where *γ* and *d* are hyperparameters. Then, covariates are introduced into the composite quantile regression single-index model

$$\pi \left(\eta_{n}|\beta ,\gamma \right)\propto det{\left[C_{n}\right]}^{-1/2}\;\text{exp}\;\{-\frac{\eta_{n}^{T}C_{n}^{-1}\eta_{n}}{2}\},$$

where ***η***_*n*_ = (*η*_1_, *η*_2_, …, *η*_*n*_)^*T*^, ***C***_*n*_ is an *n* × *n* matrix with entries

$$C\left(x_{i},x_{j}\right)=\gamma \;\text{exp}\;\left\{-{\left(x_{i}^{T}\beta -x_{j}^{T}\beta \right)}^{2}/d\right\}.$$

As noted in Gramacy and Lian [21] and Hu et al. [22], it is unnecessary to keep ‖***β***‖ = 1, so the covariance function is reformulated as

$$C\left(x_{i},x_{j}\right)=\gamma \;\text{exp}\;\left\{-{\left({x_{i}}^{T}\beta -{x_{j}}^{T}\beta \right)}^{2}\right\}.$$

Since ***β*** is not constrained to have unit norm, ***β*** can follow any prior. We choose the Gaussian prior on each component.

$$\pi \left(\beta_{j}|\sigma \right)=\frac{1}{\sqrt{2\pi \sigma }}e^{-\frac{\beta_{j}^{2}}{2\sigma }},\;\;\text{for}\;j=1,\cdots ,p.$$

To summarize, the Bayesian hierarchical formulation is provided below.

$\pi \left(y_{i}|\eta ,\alpha_{m},\beta ,e_{im}\right)∼N(\alpha_{m}+\eta \left({x_{i}}^{T}\beta \right)+\left(1-2\tau_{m}\right)e_{im},2\sigma e_{im})$ ;

$\pi \left(\eta_{n}|\gamma ,\beta \right)∼N\left(0,C_{n}\right),C_{n}\left(i,j\right)=C_{n}\left(\gamma ,\beta \right)=\gamma \;\text{exp}\;\{-{\left({x_{i}}^{T}\beta -{x_{j}}^{T}\beta \right)}^{2}\}$ ;

$\pi \left(\beta_{j}|\sigma \right)=\frac{1}{\sqrt{2\pi \sigma }}e^{-\frac{\beta_{j}^{2}}{2\sigma }}$ , *σ* > 0, *j* = 1, 2, …, *p*;

*π*(*e*_*im*_) ∼ exp(*τ*_*m*_(1−*τ*_*m*_)*σ*^−1^);

*π*(*σ*) ∼ *IG*(*a*_*σ*_, *b*_*σ*_);

*π*(*γ*) ∼ *IG*(*a*_*γ*_, *b*_*γ*_);

*π*(*α*_*m*_) ∝ 1, *m* = 1, 2, …, *M*, *i* = 1, 2, …, *n*.

The hyperpriors for *σ*, *γ* are set to be *IG*(*a*_*σ*_, *b*_*σ*_) and *IG*(*a*_*γ*_, *b*_*γ*_), where *IG* denotes the inverse Gamma distribution and Ga denotes the Gamma distribution. All of the hyperparameters *a*_*σ*_, *b*_*σ*_, *a*_*γ*_, *b*_*γ*_ are set to be 0.5 in numerical experiments. Sensitivity analysis reveals that the results are insensitive to the selection of hyperparameters.

### 2.3 MCMC sampling

In this section, we will provide the MCMC sampling procedure for model (3). The posterior distributions for all of the unknown parameters and latent variables are proportional to the joint distribution:

$$\pi \left(\beta ,\eta_{n},e_{im},\sigma ,\gamma ,\alpha_{m}|y\right)\propto \pi \left(y|\beta ,\eta ,\sigma ,\alpha_{m},e_{im}\right)\pi \left(\eta_{n}|\gamma ,\beta \right)\pi \left(\beta |\sigma \right)\pi \left(e_{im}\right)\pi \left(\sigma \right)\pi \left(\gamma \right)\pi \left(\alpha_{m}\right).$$

The posterior distributions for ***η***_*n*_ is:

$$\begin{array}{c} \eta_{n}|\beta ,\alpha ,e_{n},\gamma ,\sigma ∼N\left(\mu ,\Sigma \right), \end{array}$$ (5)

with

$$\begin{array}{ccc} \Sigma & = & C_{n}{\left(C_{n}+E\right)}^{-1}E\\ \mu & = & C_{n}{\left(C_{n}+E\right)}^{-1}EF \end{array}$$

Here, $E=\text{diag}\{{\tilde{e}}_{1}^{-1},\cdots ,{\tilde{e}}_{n}^{-1}\}$, ${\tilde{e}}_{i}=\sum_{m=1}^{M}\left(2\sigma e_{im}\right)$, and ***F*** = (*F*_1_, *F*_1_, ⋯, *F*_*n*_)^*T*^, $F_{i}=\sum_{m=1}^{M}{\left(2\sigma e_{im}\right)}^{-1}z_{im}$, *z*_*im*_ = *y*_*i*_ − *α*_*m*_ − (1 − 2*τ*_*m*_)*e*_*im*_.

Similarly, the posterior distribution of ***β***, *γ* and *α*_*m*_ are

$$\begin{array}{ccc} \pi (\beta |\alpha ,e_{n},\gamma ,\sigma ) & \propto & A\left(\beta ,\alpha ,e_{n},\gamma ,\sigma \right)\;\text{exp}\;\{-\frac{1}{2\sigma }\beta^{T}\beta \}\\ \pi (\alpha |\beta ,e_{n},\gamma ,\sigma ) & \propto & A(\beta ,\alpha ,e_{n},\gamma ,\sigma )\\ \pi (\gamma |\beta ,e_{n},\alpha_{m}) & \propto & A\left(\beta ,\alpha ,e_{n},\gamma ,\sigma \right){\left(\frac{1}{\gamma }\right)}^{a_{\gamma }+1}\;\text{exp}\;\{-\frac{1}{\gamma }b_{\gamma }\}, \end{array}$$ (6)

where

$$\begin{array}{c} A\left(\beta ,\alpha ,e_{n},\gamma ,\sigma \right)={\left|E\right|}^{1/2}{\left|C_{n}+E\right|}^{-\frac{1}{2}}\;\text{exp}\;\{-\frac{1}{2}\sum_{i=1}^{n}\sum_{m=1}^{M}\frac{z_{im}^{2}}{2\sigma e_{im}}+\frac{1}{2}\mu^{T}\Sigma^{-1}\mu \} \end{array}$$

Next, we continue to derive the full conditional distribution of *σ*, λ, *e*_*im*_, *α*_*m*_. For fixed *i*, *m*, we have

$$\begin{array}{c} e_{im}|\eta_{i},\alpha_{m}∼GIG(\frac{1}{2},\sqrt{\frac{{\left(y_{i}-\alpha_{m}-\eta_{i}\right)}^{2}}{2\sigma }},\frac{1}{\sqrt{2\sigma }}), \end{array}$$ (7)

where the probability density function of *GIG*(*ρ*, *m*, *n*) is

$$f\left(x|\rho ,m,n\right)=\frac{{\left(n/m\right)}^{\rho }}{2K_{\rho }\left(mn\right)}x^{\rho -1}\;\text{exp}\;\{-\frac{1}{2}\left(m^{2}x^{-1}+n^{2}x\right)\}.$$

Here, *x* > 0, −∞<*ρ* < ∞, *m* ≥ 0, *n* ≥ 0, *K*_*ρ*_ is the third modified Bessel function (Barndorff-Nielsen and Shephard [29]).

Finally, the posterior distributions for *σ* is

$$\begin{array}{c} \pi \left(\sigma |\beta ,\lambda \right)∼IG(\frac{Mn}{2}+\frac{p}{2}+a_{\sigma },v_{\sigma },) \end{array}$$ (8)

with $v_{\sigma }=\sum_{i=1}^{n}\sum_{m=1}^{M}\{\frac{1}{4e_{im}}{\left(z_{im}-\eta_{i}\right)}^{2}+\tau_{m}\left(1-\tau_{m}\right)e_{im}\}+\frac{1}{2\sigma }\beta^{T}\beta +b_{\sigma }$.

The details of derivation are given in S1 Appendix. The Metropolis-within-Gibbs algorithm can be used to sample from the posterior distribution. The variables $\eta_{n}^{\left(t\right)}$, $e_{im}^{\left(t\right)}$, *σ*^(*t*)^ can be directly derived based on the respective full conditional distributions. (⋅)^(*t*)^ denotes the sampled values at iteration *t*. For ***β***^(*t*)^, a Metropolis step with proposal distribution $N(\beta^{\left(t-1\right)},\sigma_{\beta }^{2}I)$ is used. For ***α***^(*t*)^, a Metropolis step with proposal distribution $N(\alpha^{\left(t-1\right)},\sigma_{\alpha }^{2}I)$ is used, and for *γ*^(*t*)^, we propose the new value from $\text{log}\gamma^{\left(t\right)}∼N\left(\text{log}\gamma^{\left(t-1\right)},\sigma_{\gamma }^{2}\right)$. In practice, *σ*_*β*_, *σ*_*α*_ and *σ*_*γ*_ control the acceptance rate to be within 10%∼30%. In the Metropolis step and Gibbs sampling, we generate 20,000 samples and burn in 10000 samples. The detailed sampling proceeds are as follows.

- The index vector ***β***, ***α*** and parameter *γ* are sampled from their posterior distributions (6) based on Metropolis steps;
- Sample the nonparametric link function *η*(⋅) from its posterior distribution (5);
- The remaining parameters (*e*_*im*_, *σ*) are sampled from their full conditional distributions (7) and (8) respectively.

## 3 Numerical illustrations

We use Monte Carlo simulations to study the performance of the proposed method (BCQR) with comparison to the Bayesian quantile regression (BQR) based on a single quantile *τ* = 0.5, and the Bayesian linear regression (BLR) for single index model.

**Example** 1 Data is generated from the following composite quantile regression for single-index model:

$$y=\eta \left(x^{T}\beta \right)+0.1\varepsilon ,\;\;\eta \left(t\right)=sin(\pi \frac{t-A}{C-A}),$$

where $\beta ={\left(\beta_{1},\beta_{2},\beta_{3},\beta_{4}\right)}^{T}=\frac{1}{\sqrt{7}}{\left(2,1,1,1\right)}^{T}$, $A=\frac{\sqrt{3}}{2}-\frac{1.645}{\sqrt{12}}$, $C=\frac{\sqrt{3}}{2}+\frac{1.645}{\sqrt{12}}$. The predictors ***x*** = (*x*_1_, *x*_2_, *x*_3_, *x*_4_)^*T*^ are uniform in [0, 1]^4^.

**Example** 2 Data is generated from the following model:

$$y=\eta \left(x^{T}\beta \right)+0.2\varepsilon ,\;\;\eta \left(t\right)=t^{2}\;\text{exp}\;\left(t\right),$$

where $\beta ={\left(\beta_{1},\beta_{2}\right)}^{T}=\frac{1}{\sqrt{5}}{\left(1,2\right)}^{T}$. The predictors ***x*** = (*x*_1_, *x*_2_)^*T*^ are uniform in [−1, 1]^2^.

**Example** 3 Data is generated from the following model:

$$y=\eta \left(x^{T}\beta \right)+0.1\varepsilon ,\;\;\eta \left(t\right)=\text{sin}\;\left(2t\right)+2\;\text{exp}\;\left(-16t^{2}\right),$$

where $\beta ={\left(\beta_{1},\beta_{2}\right)}^{T}=\frac{1}{\sqrt{5}}{\left(1,2\right)}^{T}$. The predictors ***x*** = (*x*_1_, *x*_2_)^*T*^ are uniform in [0, 1]^2^.

For all models, we consider the following four different distributions for the random error *ε*:

- (1) standard normal distribution, *N*(0, 1);
- (2) t-distribution with degrees of freedom 3, *t*(3);
- (3) exponential distribution with the rate 0.5, *Exp*(0.5);
- (4) mixture normal distribution (MN), 0.5*N*(−2, 1) + 0.5*N*(2, 1).

The sample sizes *n* = 50 and *n* = 100 are considered. Since the frequentist approach requires the identifiability constraint ‖***β***‖ = 1, we normalize the Bayesian estimates of the index vector to have unit norm, while the first component of the index vector requires to be positive to resolve the sign indeterminacy.

The biases and standard deviation (SDs × 100) calculated by BCQR_9_, BCQR_5_, BQR and BLR for different error distributions in Examples 1–3 are summarized in Tables 1–3. Here, BCQR_5_ and BCQR_9_ represent BCQR method with *M* = 5 and *M* = 9 respectively. From Table 1, it can be seen that when the error follows mixture normal distribution, the performances of four methods are similar. Moreover, when the error follows *N*(0, 1), the BLR performs the best. For example, when *n* = 100 in example 1, the biases of all parameters are similar for four methods, but the SDs of BLR are the least. For *t*(3) error distribution, the biases of BCQR_9_, BCQR_5_ and BQR perform better than BLR for most cases and the SDs of BCQR_9_, BCQR_5_ and BQR are smaller than that of BLR for four parameters with two kinds of sample sizes. Thus, the BCQR_9_, BCQR_5_ and BQR outperform BLR for *t*(3) error distribution. The BCQR_9_ and BCQR_5_ outperform BQR for the exponential error distribution, because there are still the least SDs for BCQR_9_ and BCQR_5_. From Tables 2 and 3, we can obtain similar conclusions to those in Example 1 and even the advantages of BCQR_9_ and BCQR_5_ are more obvious, that is, the biases and SDs of BCQR_9_ or BCQR_5_ are both significantly smaller than that of BLR and BQR for all settings. To sum up, we can see that the performances of BCQR with *M* = 5 and *M* = 9 are similar, which is better than the BQR and BLR method in most cases.

**Table 1:** Comparison of Bias (×100) and SDs (×100) of $\beta =\frac{1}{\sqrt{7}}{\left(2,1,1,1\right)}^{T}={\left(0.7559,0.3780,0.3780,0.3780\right)}^{T}$ for BCQR, BQR and BLR based on 100 replications in each case for simulation Example 1.

error	*n*	*β*	Bias				SD			
			BLR	BQR	BCQR_5_	BCQR_9_	BLR	BQR	BCQR_5_	BCQR_9_
N(0,1)	50	*β*_1_ = 0.7559	-0.2947	-0.1777	-0.1046	-0.0750	1.9844	2.0512	2.1127	2.1206
		*β*_2_ = 0.3780	0.0512	-0.0466	-0.0187	-0.0293	2.7410	2.6323	2.6186	2.6621
		*β*_3_ = 0.3780	-0.3107	-0.2984	-0.4147	-0.4511	2.3064	2.4198	2.3748	2.4336
		*β*_4_ = 0.3780	0.5468	0.3846	0.3266	0.3075	2.4121	2.6198	2.6209	2.6176
	100	*β*_1_ = 0.7559	-0.0307	0.1204	0.0019	-0.0019	1.0910	1.3468	1.2984	1.2772
		*β*_2_ = 0.3780	-0.2451	-0.4395	-0.2914	-0.2713	1.3046	1.5896	1.5057	1.4553
		*β*_3_ = 0.3780	0.2914	0.1455	0.2481	0.3236	1.2998	1.5754	1.4018	1.3506
		*β*_4_ = 0.3780	-0.0746	-0.0808	-0.0740	-0.1561	1.4693	1.7981	1.6299	1.5746
t(3)	50	*β*_1_ = 0.7559	-0.4822	-0.3638	-0.1861	-0.1440	2.8952	2.4040	2.4096	2.4782
		*β*_2_ = 0.3780	0.2989	0.1959	0.0926	0.0948	4.1258	3.7727	3.7278	3.6969
		*β*_3_ = 0.3780	0.2725	0.2148	-0.0175	0.0022	3.8043	2.9012	2.9203	2.9561
		*β*_4_ = 0.3780	-0.3257	-0.1924	-0.2235	-0.3317	3.8076	3.1939	3.3793	3.3490
	100	*β*_1_ = 0.7559	-0.5303	-0.3527	-0.3865	-0.3835	2.7579	1.5400	1.6667	1.6712
		*β*_2_ = 0.3780	0.7593	0.2633	0.4461	0.4561	2.4873	2.1281	2.1589	2.1806
		*β*_3_ = 0.3780	-0.1058	-0.1384	-0.1277	-0.1916	2.6405	1.9923	1.9145	1.9218
		*β*_4_ = 0.3780	0.0235	0.3786	0.2435	0.2898	2.8529	2.1066	2.2031	2.1909
Exp(0.5)	50	*β*_1_ = 0.7559	-0.7411	-0.5712	-0.0648	-0.0627	3.6071	3.1274	2.9425	2.9678
		*β*_2_ = 0.3780	0.4947	0.5699	0.3494	0.2001	4.5044	4.0312	3.4754	3.4888
		*β*_3_ = 0.3780	0.5267	0.2038	-0.3555	-0.3020	4.8805	4.3773	4.0366	4.0687
		*β*_4_ = 0.3780	-0.5468	-0.4245	-0.5272	-0.4445	4.2930	3.8070	3.6129	3.6637
	100	*β*_1_ = 0.7559	-0.4616	-0.3510	-0.2228	-0.2411	3.0120	2.1246	1.9499	1.9313
		*β*_2_ = 0.3780	-0.4559	-0.3814	-0.3636	-0.2867	3.5594	2.5150	2.3681	2.3241
		*β*_3_ = 0.3780	-0.2375	-0.0537	-0.1551	-0.1806	3.5406	2.9079	2.4266	2.4020
		*β*_4_ = 0.3780	0.9540	0.7313	0.6395	0.6331	3.8932	3.3202	3.0004	2.9661
MN	50	*β*_1_ = 0.7559	-0.5602	-1.0187	-0.1993	-0.0614	4.1935	5.2444	4.8294	4.6995
		*β*_2_ = 0.3780	-0.7521	-0.4597	-0.6317	-0.7681	4.5969	5.6220	4.9176	4.8947
		*β*_3_ = 0.3780	0.8292	0.9197	0.4759	0.3835	5.2809	6.9033	5.2425	5.2580
		*β*_4_ = 0.3780	-0.2181	-0.4264	-0.9134	-0.9584	5.2996	6.6287	5.9646	6.0457
	100	*β*_1_ = 0.7559	-0.0588	-0.5406	0.1521	0.1653	3.5508	4.8559	3.8724	3.5918
		*β*_2_ = 0.3780	-0.3970	-1.4136	-0.7727	-0.7597	4.3277	5.9736	4.3353	4.0039
		*β*_3_ = 0.3780	-0.1166	-0.0588	-0.5507	-0.4627	3.6529	5.9077	3.9175	3.8467
		*β*_4_ = 0.3780	-0.1601	0.8981	0.1512	0.1141	3.9843	5.4601	4.0689	3.8927

**Table 2:** Comparison of Bias (×100) and SD (×100) of $\beta =\frac{1}{\sqrt{5}}{\left(1,2\right)}^{T}={\left(0.4472,0.8944\right)}^{T}$ for BCQR, BQR and BLR based on 100 replications in each case for simulation Example 2.

error	*n*	*β*	Bias				SD			
			BLR	BQR	BCQR_5_	BCQR_9_	BLR	BQR	BCQR_5_	BCQR_9_
N(0,1)	50	*β*_1_ = 0.4472	2.0892	1.9277	1.0177	0.9909	3.3104	3.4374	3.2253	3.3272
		*β*_2_ = 0.8944	-1.1542	-1.0746	-0.5893	-0.5803	1.7566	1.8100	1.6537	1.7141
	100	*β*_1_ = 0.4472	0.7185	0.7260	0.5757	0.5866	2.7304	2.8399	2.7031	2.7150
		*β*_2_ = 0.8944	-0.4144	-0.4224	-0.3404	-0.3464	1.3835	1.4311	1.3543	1.3614
t(3)	50	*β*_1_ = 0.4472	2.4408	2.0705	0.1822	0.1139	5.7992	4.4293	4.3701	4.4884
		*β*_2_ = 0.8944	-1.5212	-1.2068	-0.2244	-0.1969	3.5159	2.3630	2.2160	2.2550
	100	*β*_1_ = 0.4472	1.5621	1.3064	0.7743	0.7978	3.2672	3.1869	3.2348	3.2648
		*β*_2_ = 0.8944	-0.8738	-0.7368	-0.4649	-0.4783	1.7265	1.6784	1.7013	1.7211
Exp(0.5)	50	*β*_1_ = 0.4472	2.4408	2.0705	0.1822	0.1139	5.7992	4.4293	4.3701	4.4884
		*β*_2_ = 0.8944	-1.5212	-1.2068	-0.2244	-0.1969	3.5159	2.3630	2.2160	2.2550
	100	*β*_1_ = 0.4472	1.6961	1.4900	0.8006	0.7203	3.7411	2.8278	2.7402	2.7742
		*β*_2_ = 0.8944	-0.9672	-0.8171	-0.4573	-0.4177	1.9644	1.4923	1.4263	1.4466
MN	50	*β*_1_ = 0.4472	3.3859	7.4639	1.8285	1.3978	7.5453	12.2191	8.5924	9.4871
		*β*_2_ = 0.8944	-2.2025	-5.4572	-1.4776	-1.3779	4.3194	8.7224	4.7313	5.3671
	100	*β*_1_ = 0.4472	1.5041	2.8928	0.5506	0.3649	4.8263	5.6792	4.8215	4.7192
		*β*_2_ = 0.8944	-0.9336	-1.7403	-0.4404	-0.3391	2.5486	3.0935	2.4972	2.4347

**Table 3:** Comparison of Bias (×100) and SD (×100) of $\beta =\frac{1}{\sqrt{5}}{\left(1,2\right)}^{T}={\left(0.4472,0.8944\right)}^{T}$ for BCQR, BQR and BLR based on 100 replications in each case for simulation Example 3.

error	*n*	*β*	Bias				SD			
			BLR	BQR	BCQR_5_	BCQR_9_	BLR	BQR	BCQR_5_	BCQR_9_
N(0,1)	50	*β*_1_ = 0.4472	11.5231	7.1464	1.3183	1.0937	9.0091	8.9162	5.5884	5.8111
		*β*_2_ = 0.8944	-7.5431	-4.6594	-0.8963	-0.7972	7.0110	6.5766	3.0096	3.1023
	100	*β*_1_ = 0.4472	1.9332	1.6994	0.6512	0.4625	4.1462	4.1836	4.0007	3.7491
		*β*_2_ = 0.8944	-1.1146	-0.9924	-0.4386	-0.3293	2.1776	2.1453	1.9995	1.8743
t(3)	50	*β*_1_ = 0.4472	13.1629	9.5178	1.3948	0.7825	10.3535	9.7389	7.1237	8.1412
		*β*_2_ = 0.8944	-8.9343	-6.2605	-1.0768	-0.8528	7.8928	6.7367	3.8315	3.9700
	100	*β*_1_ = 0.4472	1.6420	2.9699	0.8384	0.4016	8.8567	4.3900	4.7306	4.5613
		*β*_2_ = 0.8944	-1.4165	-1.6892	-0.5827	-0.3480	5.0448	2.4711	2.5307	2.4028
Exp(0.5)	50	*β*_1_ = 0.4472	16.8867	14.1220	2.4447	2.0166	13.5726	12.6179	9.5746	8.0655
		*β*_2_ = 0.8944	-12.8377	-10.3177	-1.9816	-1.5134	12.4621	10.9669	5.9595	4.4478
	100	*β*_1_ = 0.4472	6.2161	5.6441	2.3699	2.1488	9.8466	6.3981	4.9009	5.0753
		*β*_2_ = 0.8944	-4.1912	-3.3753	-1.3960	-1.2905	6.6811	4.0525	2.6553	2.7527
MN	50	*β*_1_ = 0.4472	15.5209	16.0216	4.3284	3.7158	14.5228	15.4393	16.9714	17.0794
		*β*_2_ = 0.8944	-12.1640	-12.8204	-5.5938	-5.4775	13.8555	14.3306	16.7672	17.8346
	100	*β*_1_ = 0.4472	6.6404	12.0468	3.4407	2.9352	12.4192	14.5658	8.5861	6.8901
		*β*_2_ = 0.8944	-4.8840	-9.1227	-2.3369	-1.8604	8.0243	11.1544	4.7192	3.5494

In addition, we also investigate the estimated accuracy of Y by bias (×100) and SD for BLR, BQR, BCQR_5_, BCQR_9_ under different error settings. It can be found from Table 4 that the performances of four methods are similar and the advantages of them are reflected in different aspects. To be more specific, the proposed BCQR_5_ and BCQR_9_ tend to have the smaller biases, while BLR and BQR may reveal the smaller SDs.

**Table 4:** Comparison of Bias (×100) and SD of Y for BCQR, BQR and BLR based on 100 replications in each case for simulation Examples 1–3.

	error	*n*	Bias				SD			
			BLR	BQR	BCQR_5_	BCQR_9_	BLR	BQR	BCQR_5_	BCQR_9_
Example1	N(0,1)	50	0.0534	-0.0698	0.0561	0.0440	0.4191	0.4284	0.4345	0.4346
		100	0.0170	-0.0558	0.0165	-0.0080	0.4197	0.4204	0.4233	0.4235
	t(3)	50	0.0988	0.3794	0.0343	0.0344	0.4019	0.4136	0.4227	0.4233
		100	0.0309	-0.1001	0.0140	-0.0330	0.4162	0.4248	0.4289	0.4294
	Exp(0.5)	50	0.0824	-3.3449	-0.0101	-0.0067	0.4034	0.4035	0.4218	0.4228
		100	0.0339	-4.6539	-0.3103	-0.2355	0.3995	0.4113	0.4202	0.4206
	MN	50	0.1148	0.2976	0.0650	0.0311	0.4060	0.4061	0.4380	0.4398
		100	0.0400	-0.0711	0.0647	-0.0451	0.4009	0.4207	0.4318	0.4326
Example2	N(0,1)	50	-0.1550	-0.5118	0.0709	-0.0494	0.7938	0.7876	0.8139	0.8140
		100	-0.0527	0.0859	-0.1040	-0.0730	0.7737	0.7740	0.7806	0.7803
	t(3)	50	-0.3210	-1.3200	-0.1089	-0.0841	0.8034	0.7793	0.8395	0.8403
		100	-0.1267	-0.0597	-0.1900	-0.1113	0.8006	0.8013	0.8131	0.8129
	Exp(0.5)	50	-0.6330	-10.2027	-0.3193	-0.3046	0.7566	0.6997	0.8139	0.8140
		100	-0.2164	-9.2839	-0.3925	-0.1805	0.7674	0.7856	0.7965	0.7954
	MN	50	-0.6911	-6.1466	-0.3457	-0.2650	0.7771	0.5757	0.8413	0.8441
		100	-0.2674	-0.4696	-0.3869	-0.2010	0.7864	0.7760	0.8166	0.8171
Example3	N(0,1)	50	-0.1006	-0.3424	0.0251	-0.0299	0.1740	0.1911	0.2056	0.2088
		100	-0.0201	-0.0649	-0.2585	-0.2815	0.1957	0.1991	0.1910	0.1977
	t(3)	50	-0.2129	-0.3294	0.0579	-0.0315	0.1561	0.1687	0.2040	0.2062
		100	-0.0230	-0.0907	-0.1069	-0.2247	0.1706	0.1921	0.1850	0.1908
	Exp(0.5)	50	-0.3146	-4.7506	-0.1001	0.0090	0.1487	0.1579	0.2048	0.2083
		100	-0.0436	-4.8156	-0.3103	-0.2489	0.1624	0.1978	0.1921	0.1981
	MN	50	-0.3774	-0.9263	-0.2118	-0.1917	0.1628	0.1607	0.2245	0.2281
		100	-0.0639	-0.4439	0.0044	-0.0326	0.1594	0.1784	0.1953	0.1991

**Fig 1:**
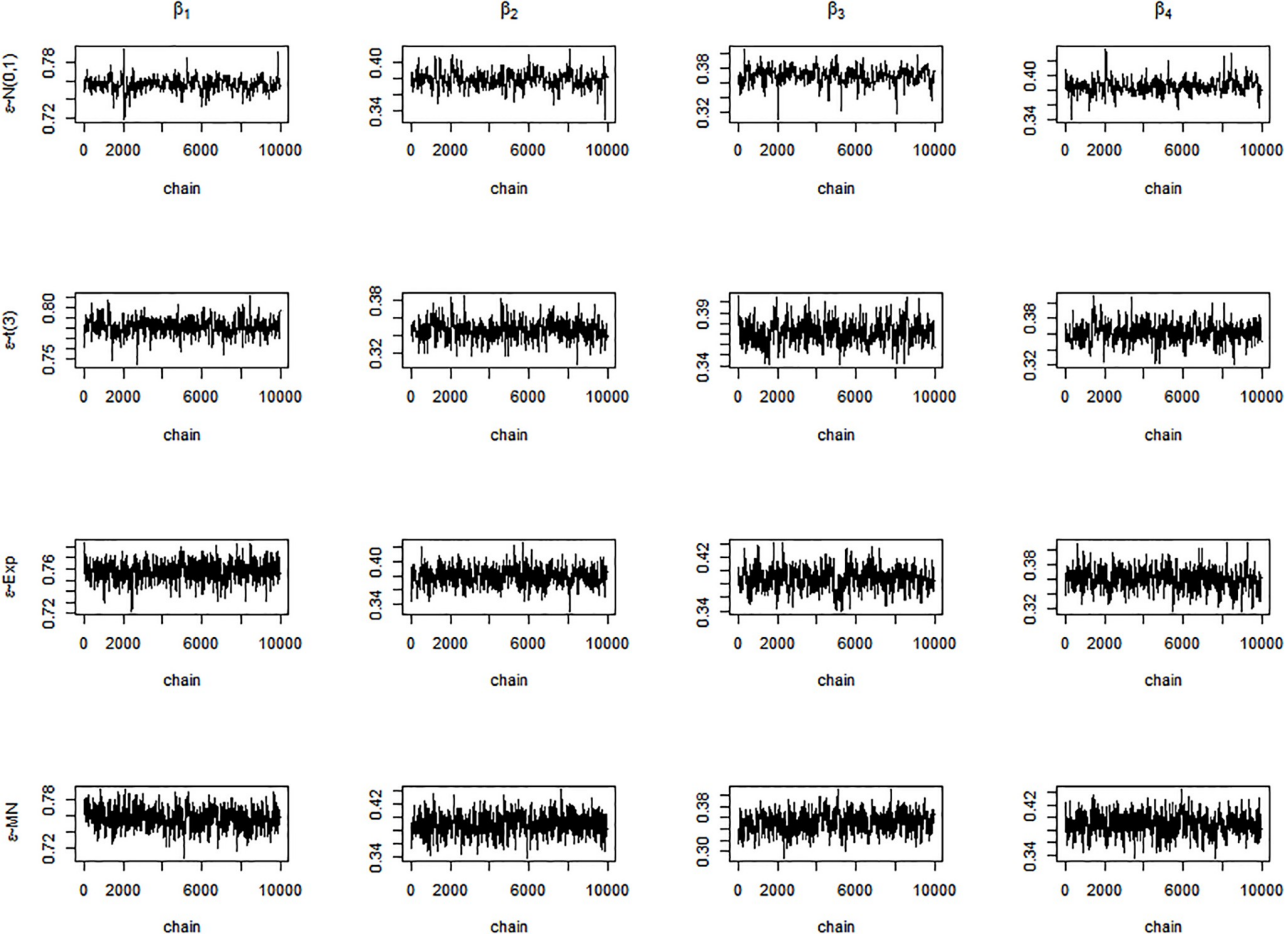
Trace plots of *β*_1_, *β*_2_, *β*_3_, *β*_4_ for BCQR_9_ in simulation Example 1 with four different error distributions, *n* = 100.

**Fig 2:**
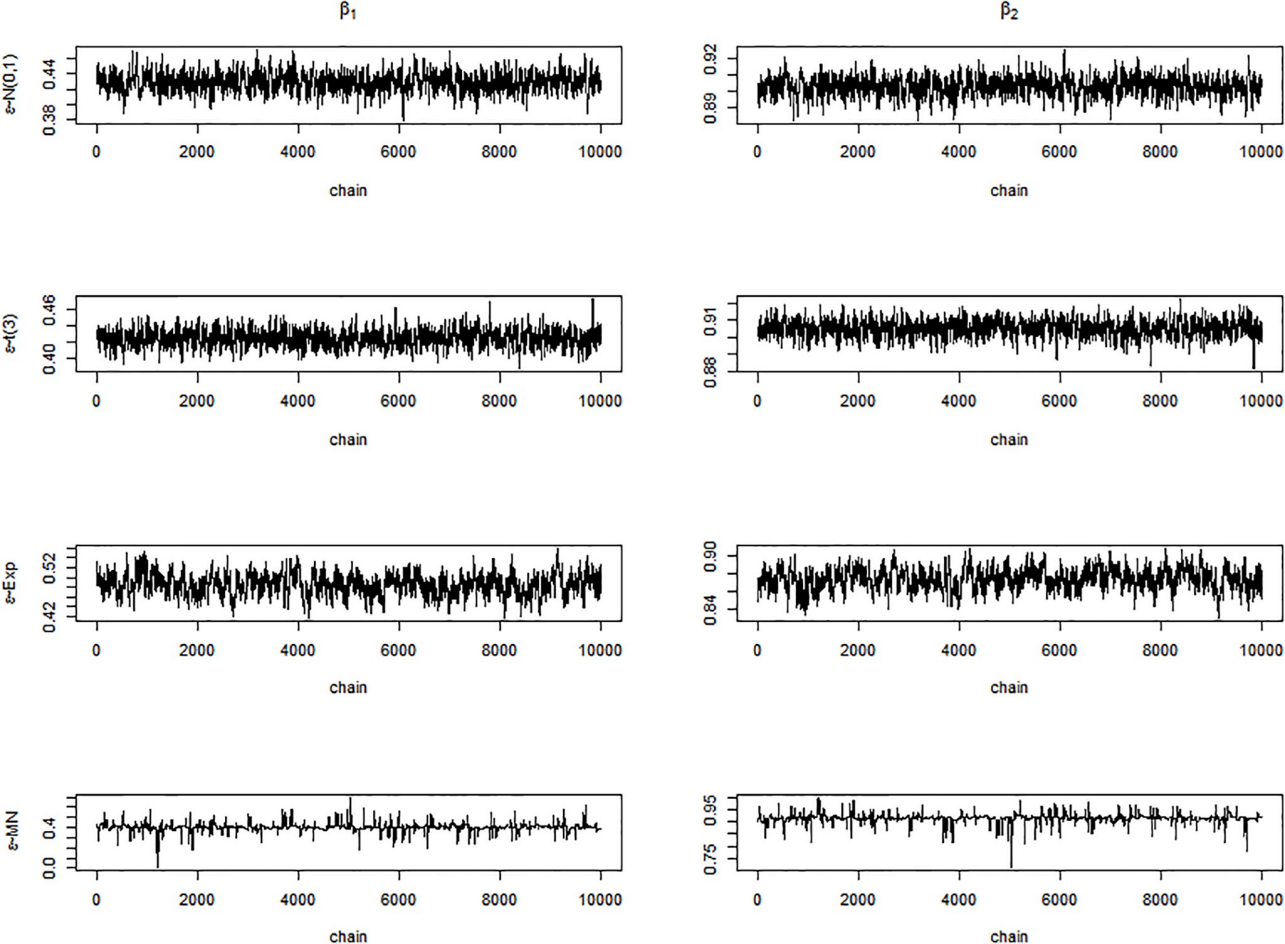
Trace plots of *β*_1_, *β*_2_, *β*_3_, *β*_4_ for BCQR_9_ in simulation Example 2 with four different error distributions, *n* = 100.

**Fig 3:**
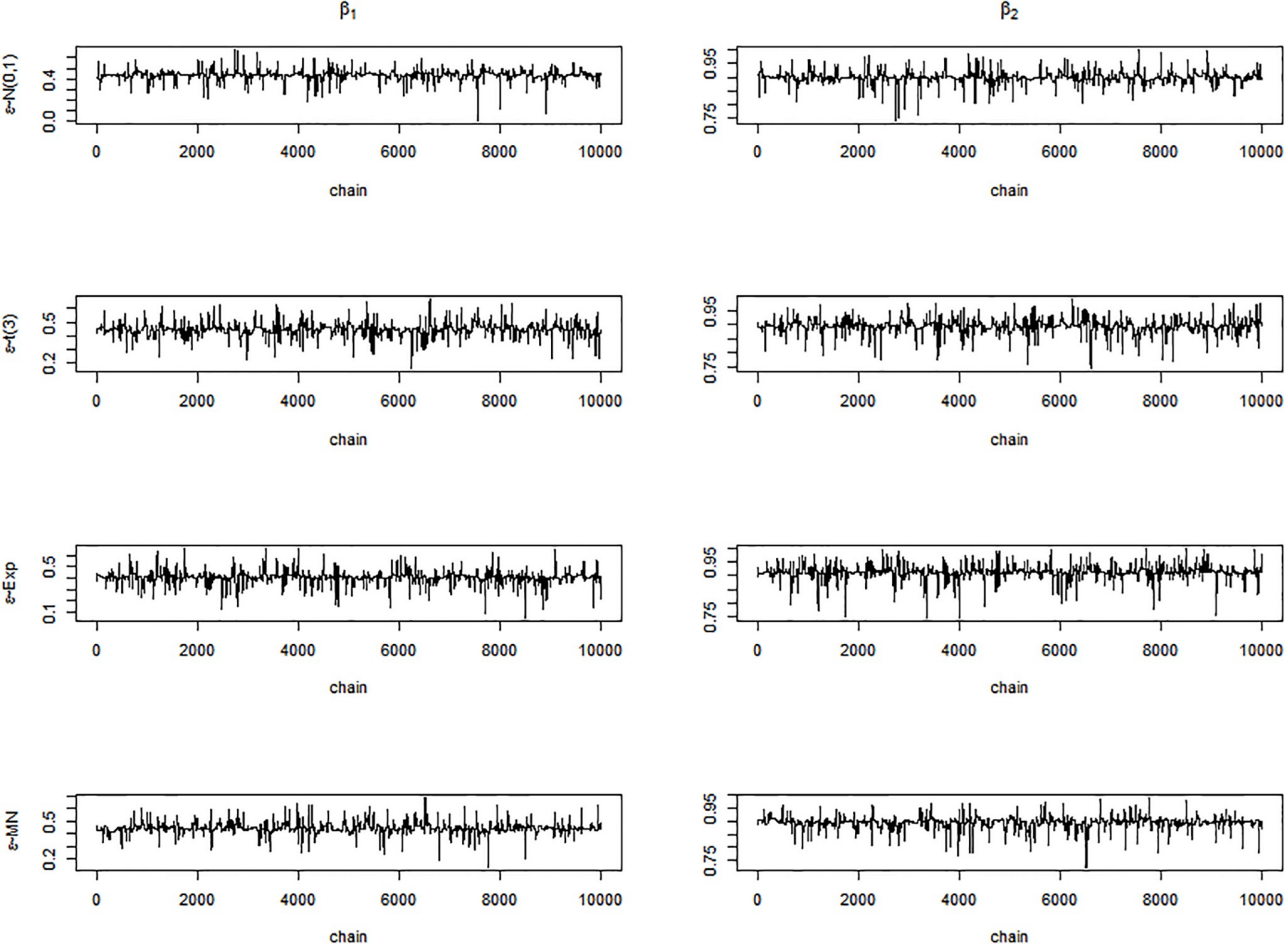
Trace plots of *β*_1_, *β*_2_, *β*_3_, *β*_4_ for BCQR_9_ in simulation Example 3 with four different error distributions, *n* = 100.

**Fig 4:**
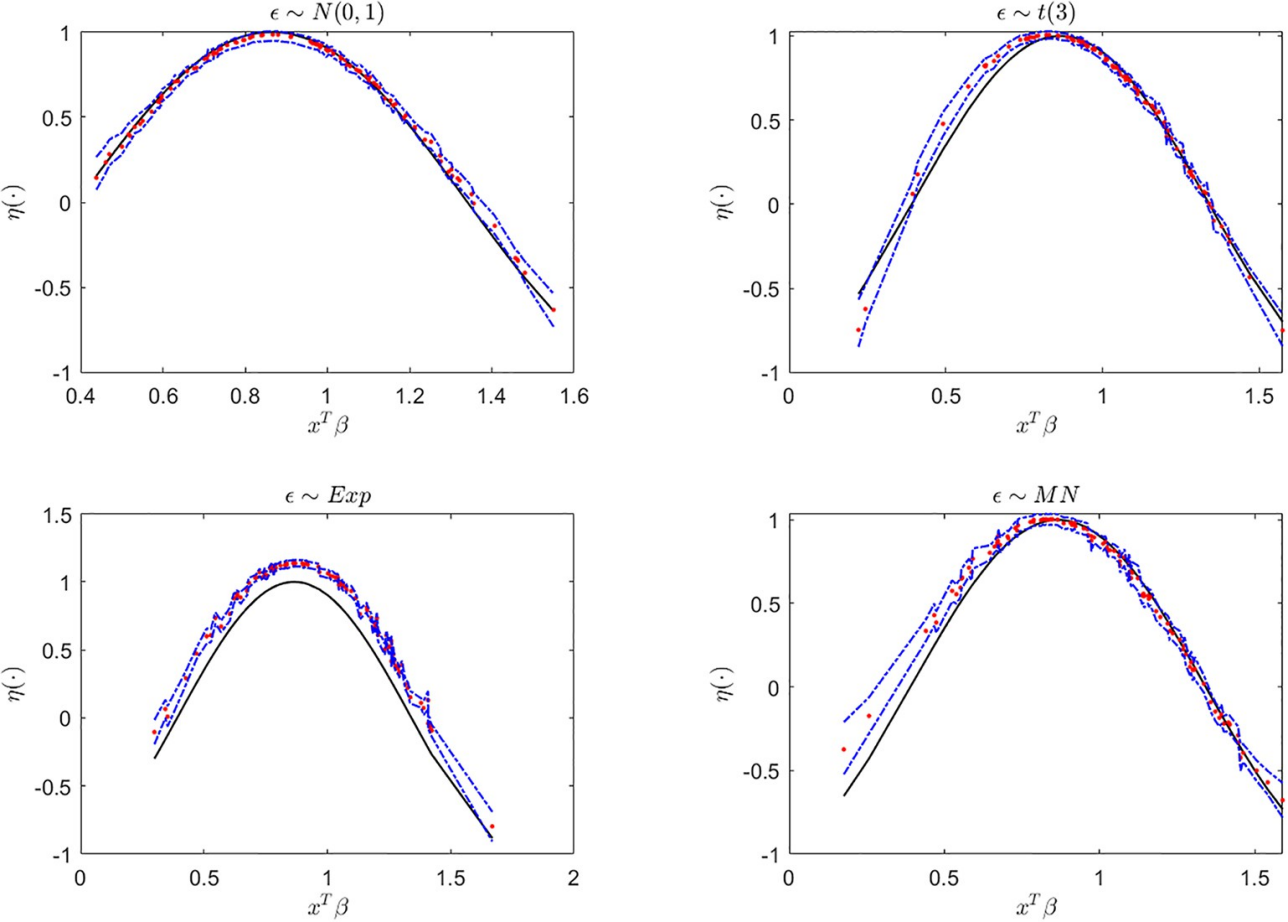
The true function (black), estimated link function (red) and CI (blue) by BCQR_9_ in simulation Example 1 with four different error distributions, *n* = 100.

**Fig 5:**
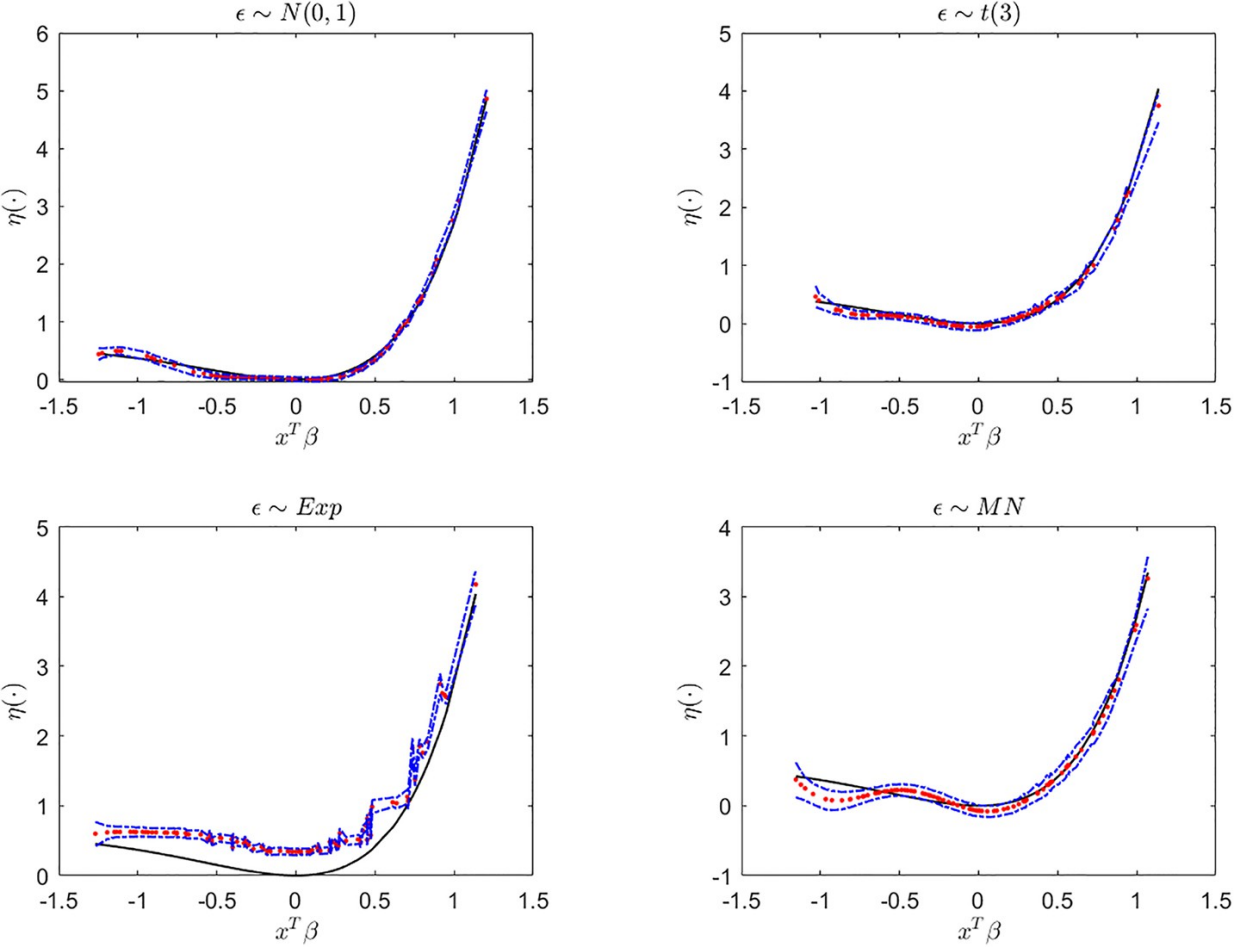
The true function (black), estimated link function (red) and CI (blue) by BCQR_9_ in simulation Example 2 with four different error distributions, *n* = 100.

**Fig 6:**
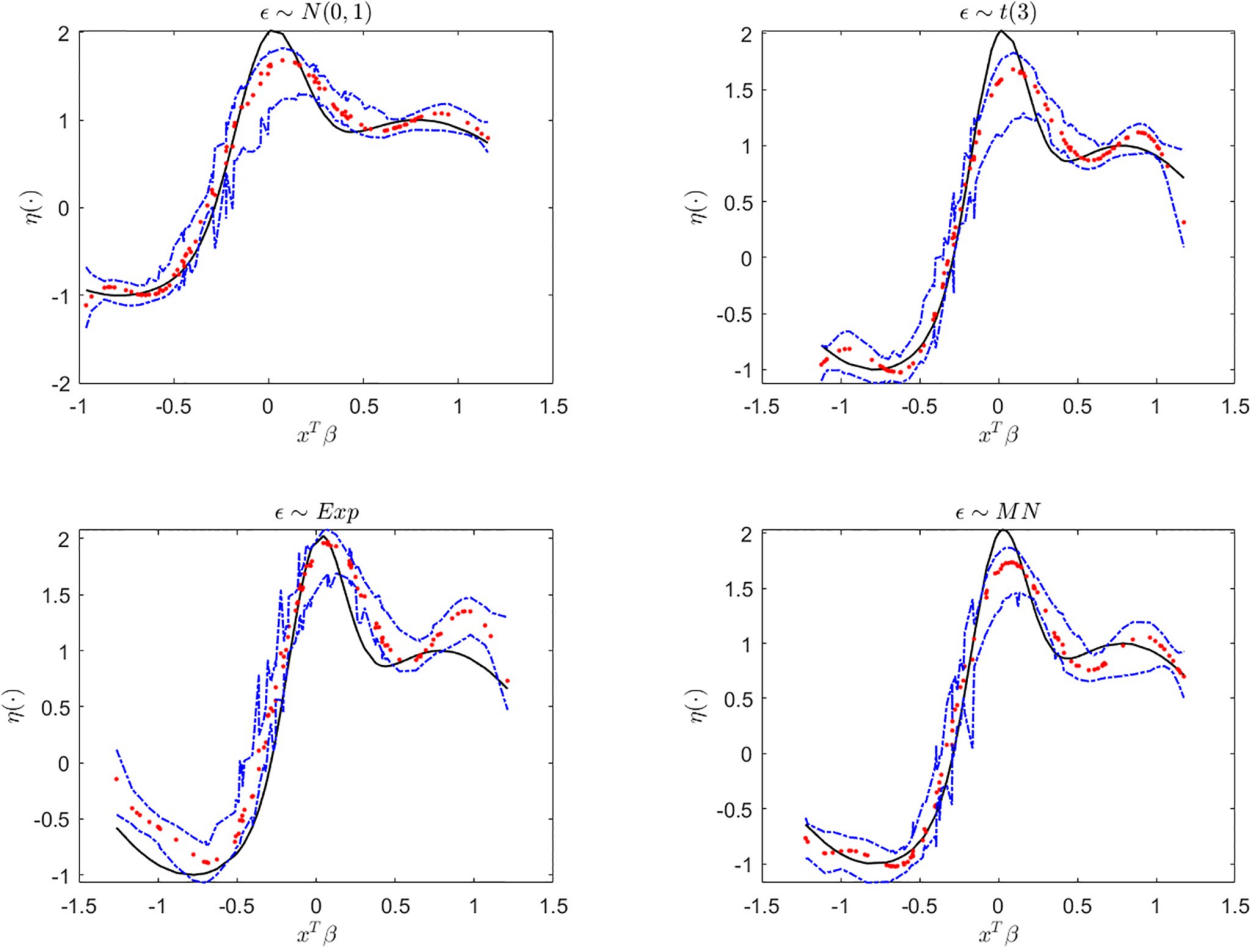
The true function (black), estimated link function (red) and CI (blue) by BCQR_9_ in simulation Example 3 with four different error distributions, *n* = 100.

Next, we display the trace-plots of some parameters to check the convergence of our approach. As the results for the four error distributions are very similar, we only show the results for BCQR_9_ with the error term *N*(0, 1) in Figs 1–3. Figs 1–3 demonstrate that the chains of posterior samples are convergent quickly, which means that the samples derived by our approach can be considered to achieve satisfactory convergence. The true function *η*(⋅) and the estimated link function by BCQR_9_ (based on posterior mean) are displayed in Figs 4–6. The 95% credible intervals (CIs) with sample size *n* = 100 are also plotted.

## 4 Real data analysis

Here, we apply the proposed approach to a body fat data set (Penrose et al. [30]), which can be founded in R package “mfp”. There are 252 observations with the response variable (Percent body fat) and 13 covariates (abdomen, weight, height, neck, chest, age, hip, thigh, knee, ankle, biceps, forearm, wrist). Liu and Yang [31] and Li et al. [32] consider the single index model variable selection procedures for this dataset. Both two methods select abdomen, neck and wrist as the significant variables. Liu et al. [15] consider the weighted composite quantile estimation of the single-index model for thise data. Similar to Liu et al. [15], we consider the response *y* = log(Percent body fat) and select the covariates: *x*_1_ = age, *x*_2_ = abdomen, *x*_3_ = wrist. One purpose of us is to estimate the relationship between body fat and other covariates. The other aim is to predict the body fat by covariates: *x*_1_ = age, *x*_2_ = abdomen and *x*_3_ = wrist. For prediction, we regard the first 150 observations as training dataset *D*_*train*_ and the rest as the testing dataset *D*_*test*_. Then, we use the coefficients obtained by training dataset to calculate the mean of the absolute prediction error (MAPE) of BQR, BCQR_5_ and BCQR_9_ based on the testing dataset *D*_*test*_, where MAPE is the mean of $\left\{|{\hat{y}}_{i}-y_{i}|,i\in D_{\text{test}\;}\right\}$.

The following model is considered:

$$y=\eta (x_{1}\beta_{1}+x_{2}\beta_{2}+x_{3}\beta_{3})+\varepsilon .$$

Before applying our methods, we exclude the observations with the percentage body fat estimated to be 0 and the density less than 1. Here, all covariates are standardized. The estimated results are summarized in Table 5.

**Table 5:** Results of Bayesian single-index model for body fat data.

		BQR	BCQR_5_	BCQR_9_
Estimate	age	0.1360	0.1266	0.1254
	abdomen	0.9574	0.9505	0.9504
	wrist	-0.2548	-0.2839	-0.2845
CI	age	(0.0490, 0.2232)	(0.0847, 0.1742)	(0.0960, 0.1654)
	abdomen	(0.9263, 0.9829)	(0.9358, 0.9634)	(0.9378, 0.9607)
	wrist	(-0.3368, -0.1529)	(-0.3267, -0.2286)	(-0.3184, -0.2517)
LCI	age	0.1742	0.0895	0.0694
	abdomen	0.0566	0.0276	0.0229
	wrist	0.1839	0.0981	0.0667
MAPE		0.3128	0.3267	0.3293

**Fig 7:**
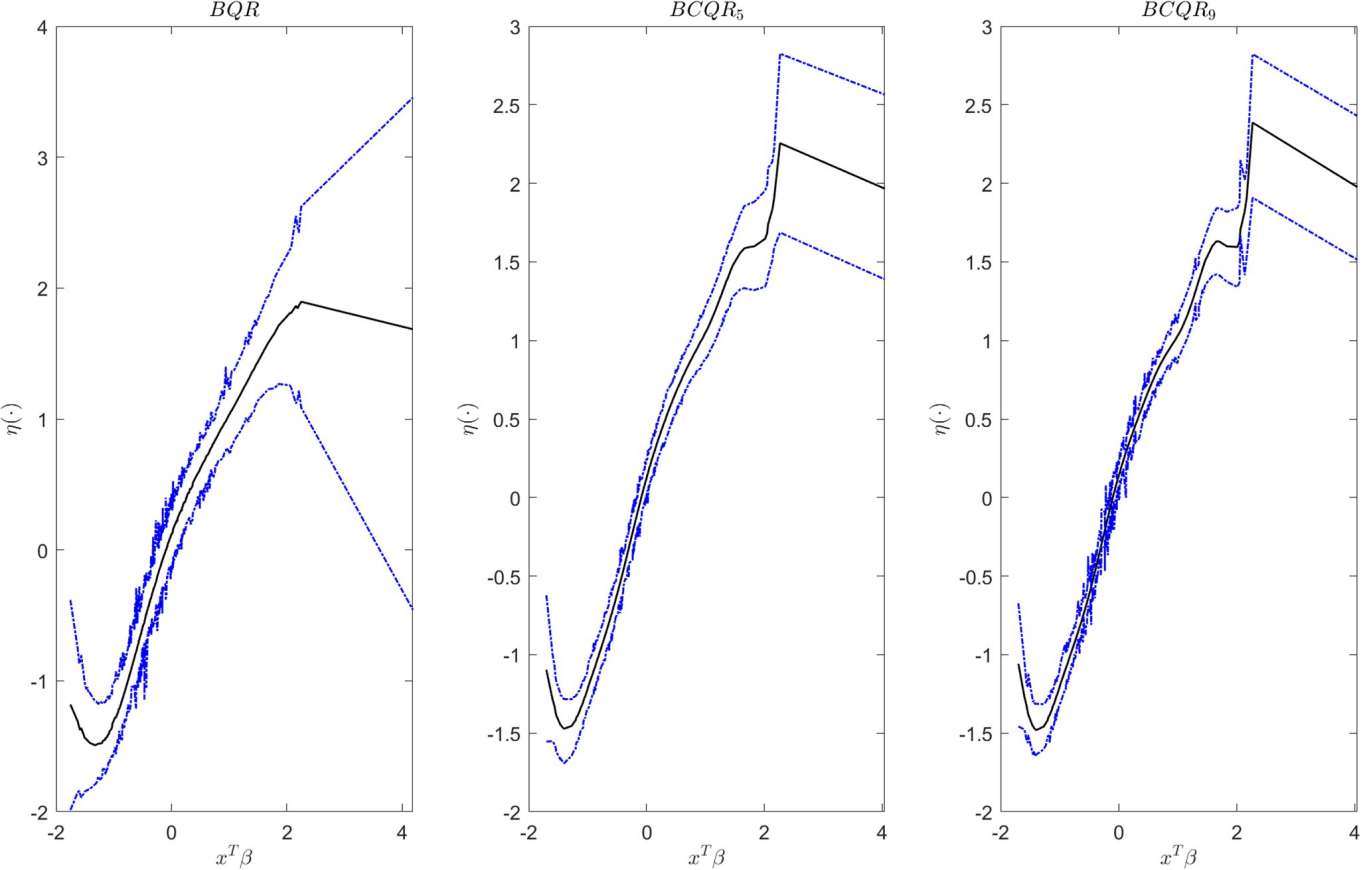
The estimated link function *η*(⋅) (black) and CI (blue) by BQR, BCQR_5_, BCQR_9_ for body fat data.

From Table 5, we can see that the age and abdomen both suggest positive relationships for response, while wrist has negative influences on the body fat. Among them, abdomen plays the greatest impact, while age has minimal effect on body fat. In addition, the estimators of ***β*** are very close to each other for BCQR_5_ and BCQR_9_. The lengthes of the confidence interval for BCQR_9_ and BCQR_5_ are both smaller than that of BQR, and the BCQR_9_ has the smallest confidence interval for ***β***. In addition, the predicted performances of three methods are also similar. Correspondingly, we also fit the link function *η*(⋅) in Fig 7. Thus, we conclude that there is a nonlinear relationship between the covariates and the response variable, which shows that the proposed method can identify the link function *η*(⋅) well.

## 5 Discussion

In this article, we propose a Bayesian composite quantile regression method for single-index models based on a Gaussian process prior for the nonparametric link function, while an efficient MCMC algorithm for posterior inference is presented. Then, we use three synthetic examples and one real dataset to illustrate and examine the performances of BCQR with comparison to BQR and BLR. The results show that the performance of proposed method is well and it can obtain a shorter length of confidence interval. The proposed method is very effective in small or median sample, while it may be less favored when the sample size goes large. This is because that the positive definiteness of the variance of the posterior distributions of the link functions may be violated for large sample size. We will study this problem in our future work. In addition, the proposed approach may be extended to the incomplete data such as censored data, missing data and so on. Also, with the progress of the times, high dimensional data is also common. Thus, our method may be added penalties such as Lasso, adaptive Lasso, SCAD to execute variable selection.

## Supporting information

S1 Appendix MCMC algorithm detailS. (PDF)

S1 Data (CSV)

## References

[1] Powell JL., Stock JH., Stoker TM. Semiparametric estimation of index coefficients. Journal of the Econometric Society. 1989; 57: 1403–1430. doi: 10.2307/1913713

[2] Härdle W., Stoker TM. Investing smooth multiple regression by the method of average derivatives. Journal of the American statistical Association. 1989; 84(408): 986–995. doi: 10.1080/01621459.1989.10478863

[3] Hardle W., Hall P., Ichimura H. Optimal smoothing in single-index models. The annals of Statistics. 1993; 21(1): 157–178. doi: 10.1214/aos/1176349020

[4] Xia Y., Härdle W. Semi-parametric estimation of partially linear single-index models. Journal of Multivariate Analysis. 2006; 97: 1162–1184. doi: 10.1016/j.jmva.2005.11.005

[5] Chen J., Gao J., Li D. Estimation in single-index panel data models with heterogeneous link functions. Econometric Reviews. 2013;32(8): 928–955. doi: 10.1080/07474938.2012.690687

[6] Zhao Y, Feng S. Robust estimation for partial linear single-index models. Journal of Nonparametric Statistics. 2022;34(1): 228–249. doi: 10.1080/10485252.2022.2027411

[7] Koenker R, Bassett JrG. Regression quantiles. Econometrica: journal of the Econometric Society. 1978;46: 33–50. doi: 10.2307/1913643

[8] Wu TZ., Yu K., Yu Y. Single-index quantile regression. Journal of Multivariate Analysis. 2010; 101(7): 1607–1621. doi: 10.1016/j.jmva.2010.02.003

[9] Lv Y., Zhang R., Zhao W., et al. Quantile regression and variable selection of partial linear single-index model. Annals of the Institute of Statistical Mathematics. 2015; 67(2): 375–409. doi: 10.1007/s10463-014-0457-x

[10] Jiang R., Qian WM. Quantile regression for single index coefficient regression models. Statistics & Probability Letters. 2016; 110: 305–317. doi: 10.1016/j.spl.2015.09.022

[11] Xu H., Fan G., Li J. Single-Index Quantile Regression with Left Truncated Data. Journal of Systems Science and Complexity. 2022; 35(5): 1963–1987. doi: 10.1007/s11424-022-1118-4

[12] Jiang R., Zhou ZG., Qian WM., et al. Single-index composite quantile regression. Journal of the Korean Statistical Society. 2012; 41(3): 323–332. doi: 10.1016/j.jkss.2011.11.001

[13] Jiang R., Zhou ZG., Qian WM., et al. Two step composite quantile regression for single-index models. Computational Statistics & Data Analysis. 2013; 64: 180–191. doi: 10.1016/j.csda.2013.03.014

[14] Jiang R., Qian WM., Zhou ZG. Weighted composite quantile regression for single-index models. Journal of Multivariate Analysis. 2016; 148:34–48. doi: 10.1016/j.jmva.2016.02.015

[15] Liu H., Yang H., Peng C. Weighted composite quantile regression for single index model with missing covariates at random. Computational Statistics. 2019; 34(4): 1711–1740. doi: 10.1007/s00180-019-00886-y

[16] Jiang R, Yu K. Single-index composite quantile regression for massive data. Journal of Multivariate Analysis. 2020; 180: 104669. doi: 10.1016/j.jmva.2020.104669

[17] Song Y., Li Z., Fang M. Robust Variable Selection Based on Penalized Composite Quantile Regression for High-Dimensional Single-Index Models. Mathematics. 2022; 10(12): 2000. doi: 10.3390/math10122000

[18] Antoniadis A., Gregoire G., McKeague I. W. Bayesian estimation in single-index models. Statistica Sinica. 2004; 14(4):1147–1164.

[19] Wang HB. Bayesian estimation and variable selection for single index models. Computational Statistics & Data Analysis. 2009; 53(7):2617–2627. doi: 10.1016/j.csda.2008.12.010

[20] Choi T., Shi JQ., Wang B. A Gaussian process regression approach to a single-index model. Journal of Nonparametric Statistics. 2011; 23(1):21–36. doi: 10.1080/10485251003768019

[21] Gramacy RB., Lian H. Gaussian process single-index models as emulators for computer experiments. Technometrics. 2012; 54(1):30–41. doi: 10.1080/00401706.2012.650527

[22] Hu Y., Gramacy RB., Lian H. Bayesian quantile regression for single-index models. Statistics and Computing. 2013; 23(4):437–454. doi: 10.1007/s11222-012-9321-0

[23] Liu CS., Liang HY. Bayesian analysis in single-index quantile regression with missing observation. Communications in Statistics-Theory and Methods. 2022; 1–29. doi: 10.1080/03610926.2022.2050399

[24] Zou H., Yuan M. Composite quantile regression and the oracle model selection theory. The Annals of Statistics. 2008; 36:1108–1126. doi: 10.1214/07-AOS507

[25] Yu K., Moyeed RA. Bayesian quantile regression. Statistics & Probability Letters. 2011; 54(4):437–447. doi: 10.1016/S0167-7152(01)00124-9

[26] Geraci M., Bottai M. Quantile regression for longitudinal data using the asymmetric Laplace distribution. Biostatistics. 2007; 8(1):140–154. doi: 10.1093/biostatistics/kxj039 16636139

[27] Luo Y., Lian H., Tian M. Bayesian quantile regression for longitudinal data models. Journal of Statistical Computation and Simulation. 2001;82(10-12):1635–1649.

[28] Kozumi H., Kobayashi G. Gibbs sampling methods for Bayesian quantile regression. Journal of statistical computation and simulation. 2011;81(11-12):1565–1578. doi: 10.1080/00949655.2010.496117

[29] Barndorff-Nielsen OE., Shephard N. Non-Gaussian OrnsteinUhlenbeck-based models and some of their uses in financial economics. Journal of the Royal Statistical Society: Series B (Statistical Methodology). 2011;63(2):167–241. doi: 10.1111/1467-9868.00282

[30] Penrose K., Nelson A., Fisher A. Generalized body composition prediction equation for men using simple measurement techniques. Medicine & Science in Sports & Exercise. 1985;17:189. doi: 10.1249/00005768-198504000-00037

[31] Liu H., Yang H., Xia X. Robust estimation and variable selection in censored partially linear additivemodels. Journal of the Korean Statistical Society. 2017; 46:88–103. doi: 10.1016/j.jkss.2016.07.002

[32] Li J., Li Y., Zhang R. B spline variable selection for the single index models. Statistical Papers. 2017; 58:691–706. doi: 10.1007/s00362-015-0721-z

