# Supplementary material for: Bayesian composite quantile regression for the single-index model
Source: PLoS One. 2023 May 10;18(5):e0285277. doi: 10.1371/journal.pone.0285277 (PMC10171657; doi:10.1371/journal.pone.0285277)
Supplement: S1 Appendix — (PDF) [file pone.0285277.s001.pdf]

Appendix: MCMC algorithm details

The posterior distributions for all unknown parameters and latent variables are proportional to the joint distribution

$$\pi(\boldsymbol{\beta}, \boldsymbol{\eta}_n, e_n, \sigma, \gamma, \boldsymbol{\alpha} \mid \boldsymbol{y}) \propto \pi(\boldsymbol{y} \mid \boldsymbol{\beta}, \eta, \sigma, \alpha_m)\pi(\boldsymbol{\eta}_n \mid \gamma, \boldsymbol{\beta})\pi(\boldsymbol{\beta} \mid \sigma)\pi(e_n)\pi(\sigma)\pi(\gamma)\pi(\alpha_m).$$

Let $z_{im} = y_i - \alpha_m - (1 - 2\tau_m)e_{im}$. The full conditional distribution of $\boldsymbol{\eta}_n$ is

$$\begin{aligned}
\pi(\boldsymbol{\eta}_n \mid \boldsymbol{\beta}, \boldsymbol{e}_n, \boldsymbol{\alpha}, \gamma, \sigma) &\propto \pi(\boldsymbol{y} \mid \alpha, \boldsymbol{\beta}, e_n)\pi(\boldsymbol{\eta}_n \mid \gamma, \boldsymbol{\beta}) \\
&\propto \exp\left\{ -\frac{1}{2} \sum_{i=1}^{n} \sum_{m=1}^{M} \frac{1}{2\sigma e_{im}} (z_{im} - \eta_i)^2 - \frac{1}{2} \boldsymbol{\eta}_n^T \boldsymbol{C}_n^{-1} \eta_n \right\} \\
&\propto \exp\left\{ -(\boldsymbol{\eta}_n - \boldsymbol{\mu})^T \boldsymbol{\Sigma}^{-1} (\boldsymbol{\eta}_n - \boldsymbol{\mu}) \right\} \\
&\sim N(\boldsymbol{\mu}, \boldsymbol{\Sigma})
\end{aligned}$$

with

$$\begin{aligned}\boldsymbol{\Sigma} &= \boldsymbol{C}_n(\boldsymbol{C}_n+\boldsymbol{E})^{-1}\boldsymbol{E}, \\ \boldsymbol{\mu} &= \boldsymbol{C}_n(\boldsymbol{C}_n+\boldsymbol{E})^{-1}\boldsymbol{E}\boldsymbol{F}.\end{aligned}$$

Here, $\boldsymbol{E} = \mathrm{diag}\{\tilde{e}_1^{-1}, \cdots, \tilde{e}_n^{-1}\}$, $\tilde{e}_i = \sum\limits_{m=1}^{M}(2\sigma e_{im})$, and $\boldsymbol{F} = (F_1, F_1, \cdots, F_n)^T$, $F_i = \sum_{m=1}^{M}(2\sigma e_{im})^{-1} z_{im}$.

The full conditional distribution of $\boldsymbol{\beta}$ is

$$\begin{aligned}\pi(\boldsymbol{\beta}|\sigma, e_n, \alpha, \gamma) &\propto \int \pi(y \mid \alpha, \boldsymbol{\beta}, e_n)\pi(\boldsymbol{\eta}_n \mid \gamma, \boldsymbol{\beta})\mathrm{d}\boldsymbol{\eta}_n \times \pi(\boldsymbol{\beta} \mid \sigma) \\ &\propto A(\boldsymbol{\beta}) \times \pi(\boldsymbol{\beta} \mid \sigma),\end{aligned}$$

where

$$A(\boldsymbol{\beta}, \boldsymbol{\alpha}, \boldsymbol{e}_n, \gamma, \sigma) = |\boldsymbol{E}|^{1/2}|\boldsymbol{C}_n + \boldsymbol{E}|^{-\frac{1}{2}} \exp\left\{-\frac{1}{2}\sum_{i=1}^{n}\sum_{m=1}^{M}\frac{z_{im}^2}{2\sigma e_{im}} + \frac{1}{2}\boldsymbol{\mu}^T\boldsymbol{\Sigma}^{-1}\boldsymbol{\mu}\right\}.$$

Similarly, we can write the posterior distributions for γ and $\boldsymbol{\alpha}$

$$\begin{aligned}\pi(\boldsymbol{\alpha} \mid \boldsymbol{\beta}, \boldsymbol{e}_n, \gamma, \sigma) &\propto A(\boldsymbol{\beta}, \boldsymbol{\alpha}, \boldsymbol{e}_n, \gamma, \sigma) \\ \pi(\gamma \mid \boldsymbol{\beta}, \boldsymbol{e}_n, \alpha_m) &\propto A(\boldsymbol{\beta}, \boldsymbol{\alpha}, \boldsymbol{e}_n, \gamma, \sigma)\left(\frac{1}{\gamma}\right)^{a_\gamma+1}\exp\left\{-\frac{1}{\gamma}b_\gamma\right\},\end{aligned}$$

Next, we derive the full conditional distributions of σ and e_{im}. For fixed i, m, we have

$$\begin{aligned}&\pi\left(e_{im} \mid \eta_i, \gamma, \alpha_m\right) \\ &\propto e_{im}^{-\frac{1}{2}} \exp\left\{-\frac{1}{4\sigma e_{im}}\left[y_i - \alpha_m - \eta_i - (1-2\tau_m)\, e_{im}\right]^2\right\}\pi\left(e_{im}\right) \\ &\propto e_{im}^{-\frac{1}{2}} \exp\left\{-\frac{1}{2}e_{im}^{-1}\left[\frac{1}{2}\left(y_i - \alpha_m - \eta_i - (1-2\tau_m)\, e_{im}\right)^2\right] - \frac{\tau_m\left(1-2\tau_m\right)}{\sigma}e_{im}\right\} \\ &\propto e_{im}^{-1/2} \exp\left\{-\frac{1}{2}\left[\frac{(y_i - \alpha_m - \eta_i)^2}{2\sigma}e_{im}^{-1} + \frac{1}{2\sigma}e_{im}\right]\right\} \\ &\sim GIG\left(\frac{1}{2}, \sqrt{\frac{(y_i - \alpha_m - \eta_i)^2}{2\sigma}}, \frac{1}{\sqrt{2\sigma}}\right),\end{aligned}$$

The posterior distributions for σ is

$$\pi(\sigma \mid \boldsymbol{\beta}, \lambda) \sim IG\left(\frac{Mn}{2} + \frac{p}{2} + a_\sigma, v_\sigma,\right)$$

with $v_\sigma = \sum_{i=1}^{n}\sum_{m=1}^{M}\left\{\frac{1}{4e_{im}}(z_{im} - \eta_i)^2 + \tau_m(1-\tau_m)e_{im}\right\} + \frac{1}{2\sigma}\boldsymbol{\beta}^T\boldsymbol{\beta} + b_\sigma$.
